# Assessment of Women’s Sexual Function and Contributing Factors in Tunisia

**DOI:** 10.1192/j.eurpsy.2025.2304

**Published:** 2025-08-26

**Authors:** A. Hadj Salah, N. Faouel, I. Batbout, M. Ben Mbarek, A. Haouala, F. Zaafrane, A. Mhalla, B. Amamou

**Affiliations:** 1 psychiatry, Fattouma Bourguiba Hospital, Monastir, Tunisia

## Abstract

**Introduction:**

Sexual function is essential to women’s health, impacting psychological well-being and relationships. In Tunisia, cultural norms may influence how sexual dysfunction is reported and perceived. This study examines the prevalence of sexual dysfunction and its psychological consequences.

**Objectives:**

To evaluate sexual function in Tunisian women and assess its correlation with psychological distress.

**Methods:**

This cross-sectional descriptive study was conducted in March 2023 with 80 Tunisian women, aged 24-50, using a self-administered online questionnaire. Data collection involved the Female Sexual Function Index (FSFI) to assess sexual dysfunction and the Depression Anxiety and Stress Scale (DASS-21) for psychological well-being. Key sociodemographic variables, including marital status, number of children, and socio environmental context were also collected.

**Results:**

We gathered data from 80 women out of 500 distributed questionnaires (16%).

Participants had a mean age of 33.35 years. A majority (63.7%) had children, and 93.8% resided in urban areas. Notably, the majority of women who responded to the questionnaire (74%) were married.

Among the study population, the median FSFI score was 23.65, with 61.3% scoring below the threshold of 26.55, indicating sexual dysfunction. For married women (n=59), the mean FSFI score was 23.7 ± 7.9. The detailed FSFI scores for the entire population and married women are presented in Table I, indicating that the most affected domains of sexual function were excitation and desire. Severe depression, anxiety, and stress were reported by 6.3%, 12.5%, and 6.3% of participants, respectively.

Sexual dysfunction was significantly associated with depression (p=0.02). However, no statistically significant associations were found between anxiety, stress, and sexual dysfunction.

**Table:** 

FSFI Domain	Overall Score Mean ± SD	Married Women Score Mean ± SD
Desire	**3.5 ± 1.31**	**3.7 ± 1.10**
Excitation	**3.8 ± 2.10**	**4.1 ± 1.56**
Lubrification	**3.6 ± 2.12**	**4.4 ± 1.48**
Orgasm	**3.38 ± 2.12**	**4.2 ± 1.80**
Satisfaction	**3.38 ± 2.26**	**4.2 ± 1.80**
Pain	**2.5 ± 1.51**	**3.0 ± 1.10**
Total FSFI	**19.79 ± 10.66**	**23.7 ± 7.9**

**
Table I: FSFI Scores among the Study Population**

**Conclusions:**

The study shows a high prevalence of sexual dysfunction among Tunisian women, tied to psychological distress, mainly depression. Enhancing sexual health and mental well-being is key to improving overall quality of life and addressing marital issues. Future research should explore culturally sensitive interventions to enhance sexual health and support women’s mental well-being.

**Disclosure of Interest:**

None Declared

